# Checkpoint Inhibitor Colitis With Superimposed Clostridioides difficile Infection

**DOI:** 10.7759/cureus.37006

**Published:** 2023-04-01

**Authors:** Al Wahadneh Mohammad, Alexander Kusnik, Mostafa Reda Mostafa, Josenia Tan, Andrej Strapko

**Affiliations:** 1 Department of Internal Medicine, Unity Hospital, Rochester, USA; 2 Department of Pathology, Unity Hospital, Rochester, USA

**Keywords:** checkpoint inhibitor diarrhea, immune-mediated diarrhea and colitis, anti-pd-1 antibody, clostridioides difficile infection, immune-checkpoint inhibitors

## Abstract

Immune checkpoint inhibitors (ICI) are commonly used for various malignancies. A particular checkpoint inhibitor is the anti-PD-1 antibody pembrolizumab. Immune-mediated diarrhea and colitis (IMDC) is the most frequently observed immune-related adverse event (irAE) involving the gastrointestinal system. Although immune-mediated colitis precipitated by pembrolizumab is rarely life-threatening, it often necessitates a detailed diagnostic workup, including stool studies, imaging, and colonoscopy, to establish an accurate diagnosis. The coexistence of IMDC and Clostridioides difficile infection is not well understood, but patients undergoing pembrolizumab treatment have comparable risk factors to those who develop C. difficile infection. We report a case of a 76-year-old female with nonmetastatic non-small cell lung cancer who was diagnosed with IMDC responsive to steroid treatment but later developed worsening diarrhea leading to a diagnosis of checkpoint inhibitor colitis with superimposed C. difficile infection.

## Introduction

Immune checkpoint inhibitors (ICIs) have transformed cancer treatment but have been linked to immune-mediated diarrhea and colitis (IMDC) [1]. In addition, Clostridioides difficile may colonize or infect the colon; clinical manifestations vary from an asymptomatic carrier to fulminant and life-threatening colitis.

There is limited information about the incidence of C. difficile infection (CDI) in patients undergoing therapy with ICI. Our case highlights the diagnostic challenge of a 76-year-old female receiving pembrolizumab for non-small cell lung cancer (NSCLC), who presented with diarrhea and abdominal pain. Initially diagnosed with immune-mediated colitis, responsive to steroids, she was subsequently diagnosed with CDI, necessitating vancomycin treatment. A further extended course of high-dose steroids finally resulted in the resolution of her symptoms.

## Case presentation

A 76-year-old female with nonmetastatic NSCLC treated with radiotherapy and pembrolizumab presented to the emergency department (ED) for abdominal cramping and diarrhea persistent for six weeks. She received pembrolizumab every six weeks as a 400 mg infusion and finished three cycles when she started having increasingly loose bowel movements. She reported increased bowel movements to 8-12 watery bowel movements per day, associated with tenesmus, stool incontinence, and post-defecation hematochezia. She denied recent travel, sick contacts, or use of nonsteroidal anti-inflammatory drugs, proton pump inhibitors, or antibiotics.

**Figure 1:**
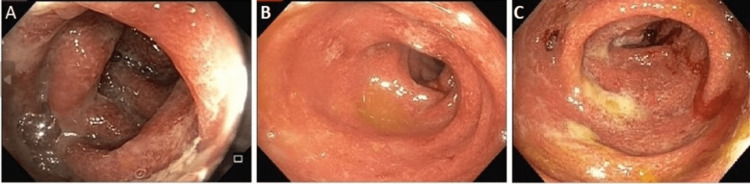
Colonoscopy indicate significant adherent mucus and exudates throughout the colon. Furthermore, diffuse mild to moderate colitis with erythema, edema, and loss of vascular marking with granular appearing mucosa. (A) Descending colon; (B) Sigmoid colon; (C) Transverse colon

Computed tomography (CT) of the abdomen and pelvis indicated pancolitis; the reading radiologist raised suspicion for checkpoint inhibitor-induced inflammatory colitis. No stool studies were collected during the ED visit as the patient had no bowel movements and was eager to be discharged. The patient received one dose of 60mg intravenous methylprednisolone and was subsequently discharged with 60mg oral prednisone to taper with a close follow-up with her gastroenterologist as she was unwilling to stay in the hospital. Additionally, a close follow-up with her oncologist was arranged in the outpatient setting. The frequency of her diarrhea improved within the following days to only three to five bowel movements daily. Her pembrolizumab was held for the time being until further workup was conducted. A colonoscopy was discussed but ultimately declined by the patient as her symptoms had already improved. Eight days later, she developed worsening diarrhea and abdominal pain and presented to the ED. Blood work indicated mild leukocytosis of 10.3/mm^3^ and mild elevation in CRP and ESR. Colonoscopy showed pancolitis with adherent mucus and exudates (Figures 1A-1C), and no ulcers were visualized. Macroscopically, diffuse moderate active colitis was demonstrated. Stool studies were obtained and resulted only positive for C. difficile. She was started on oral vancomycin 250 mg four times daily, and oral steroids were discontinued.

**Figure 2:**
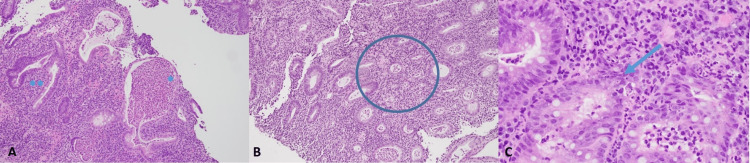
Histology of colonic mucosa The biopsy material demonstrates active colitis in the form of epithelitis, cryptitis, and frequent crypt abscesses (A*). In addition, there is focal crypt architectural distortion noted (A**). The lamina propria is expanded by chronic lymphoplasmacytic inflammation and neutrophilic infiltration (A-C). There is focal crypt atrophy (B circled) and rare foci showing apoptotic bodies (C arrow). There are no granulomas. There is no dysplasia or malignancy seen.

Histologically, active colitis was illustrated in the form of epithelitis, cryptitis, and frequent crypt abscesses. Focal crypt architectural distortion was noted with the expansion of the lamina propria by chronic lymphoplasmacytic inflammation and neutrophilic infiltration without granulomas (Figures 2A-2C).

Furthermore, focal crypt atrophy was present with rare foci showing apoptotic bodies. The findings favored immunotherapy-related colitis, but an inflammatory or infectious condition could not be entirely ruled out, given the overlapping histologic features. As she still had significant diarrhea with up to eight bowel movements daily on vancomycin, an extended methylprednisolone tapering dose was added for a suspected contributory checkpoint inhibitor colitis. On subsequent follow-up, her symptoms improved.

## Discussion

Immunotherapy using checkpoint inhibitors (ICI) is widely used to treat a variety of neoplasms [2]. The most notable role of this pathway is to promote self-tolerance and reduce autoimmunity, mainly by decreasing cytotoxic T-cell activity [2]. IMDC is the most frequently observed immune-related adverse event (irAE) involving the gastrointestinal system [3,4]. Albeit immune-mediated colitis precipitated by pembrolizumab is seldom deadly [5], the increased use of anti-PD-1 medication for various tumors will likely result in a higher frequency of reported side effects [6,7]. The currently reported incidence of diarrhea and colitis with anti-PD-1 medication is estimated at around 13% and 1.5%, respectively [8].

As immune-mediated diarrhea is similar in presentation to other causes of diarrhea, it is vital to establish an adequate diagnosis to initiate appropriate treatment promptly. Current guidelines recommend stool studies, including C. difficile, particularly in patients with severe symptoms [9]. A CT abdomen indicated pancolitis, which could have been attributed to CDI and/or IMDC, although IMDC is more frequently seen on the left side of the colon.

The Common Terminology Criteria for Adverse Events version 5 can be used to grade the severity of IMDC. The frequency of bowel movements or symptomatology is used to grade the severity of adverse events on a scale from 1 to 5. Our patient had, on initial presentation, 8-12 bowel movements daily, consistent with grade 3 severity. However, stool studies were not obtained as the patient could not have a bowel movement in the ED. Furthermore, the patient was adamant about being discharged, which complicated the proper assessment of her diarrhea. Moreover, she declined a colonoscopy as her symptoms were already improving with steroids.

Nevertheless, an infectious cause of diarrhea should have been excluded before empiric treatment with steroids, especially since a differentiation between CDI and IMDC is otherwise impossible. Furthermore, patients receiving pembrolizumab often share the same risk factors as patients with CDI infection, such as antibiotic use and frequent hospitalizations [10]. Our patient did not have the classical risk factors for CDI including recent use of proton pump inhibitors or antibiotics. There were no recent prolonged hospitalizations. A colonoscopy with biopsy performed during the second presentation indicated non-specific findings suggestive of IMDC, including active colitis mainly in the form of frequent crypt abscesses, lymphoplasmacytic inflammation, and neutrophilic infiltration of the lamina propria with crypt atrophy, and rare foci showing apoptotic bodies. Histologically, although these features overlap with an inflammatory or infectious process, IMDC must be highly considered in a patient undergoing treatment with ICI. It must be noted that certain clinical findings suggest IMDC, e.g., findings of ulcers and involvement of the left colon; more than one-third of patients can have normal colonoscopies despite clinical findings suggestive of IMDC.

Little is known about the concurrence of CDI and immune-mediated diarrhea. Reported cases illustrate a prolonged time needed to accurately diagnose and treat this type of coinfection, as seen in our case [11,12]. A retrospective single-center study explored the attributes of CDI in patients undergoing therapy with ICI in the presence of IMDC. The study concluded that the concurrence of CDI and IMDC is associated with prolonged symptom duration (20 vs. 5 days, p=0.003) and a reported higher rate of grade 3-4 diarrhea (41% vs. 7%, p=0.033) [13]. Similarly, in our case, the patient was started on treatment for the suggested IMDC with high-dose steroids. It remains speculative to what degree an active CDI was present at the time of initial presentation, as no stool studies were collected. Although the initial response to steroids favors IMDC, a few case reports highlight beneficial properties in some instances of CDI [14]. As the diarrhea started to worsen again after 7-10 days with ongoing steroid treatment, further diagnostic testing confirmed the diagnosis of CDI. But as treatment with vancomycin alone did not improve the patient’s diarrhea, steroid treatment was reinitiated, resulting in the resolution of the patient’s diarrhea.

## Conclusions

The current literature is limited regarding the concurrence of CDI and immune-mediated diarrhea. Non-specific endoscopy findings in immune-mediated diarrhea and frequent colonization of C. difficile complicate the concurrent diagnosis. Concomitant treatment with oral antibiotics and steroids might be necessary in cases in which both conditions are present.
